# Current News and Opinion

**Published:** 1885-03

**Authors:** 


### EXPANSION OF ZINC.

Zinc has the greatest degree of expansion of any of the metals. A bar nine inches long will expand to 9.026 when heated from 32° to 212°, and in proportion for intermediate amounts of change in temperature. It melts at 740° Fahr.

### Current News and Opinion.

### OBITUARY.

#### LOUIS ELSBERG, M. D.

The best of the Laryngologists in America, a scientist of high repute, a liberal, kind-hearted man, a faithful friend, closed his eyes forever, on February 19, 1885, at his residence, 614 Fifth avenue, New York. He was born April 2, 1836, at Iserlohn, Prussia, and as a boy was brought over to America by his family, which settled in Philadelphia. Here he received a regular college education and graduated an M. D. when only 21 years of age, in the Jefferson Medical College, June, 1858. Laryngology became a settled science through the re-discovery of the Laryngoscope, by the late Louis Fuerch of Vienna. At that time Elsberg was in Vienna, at the cradle of the novel specialty, and both here and in Berlin obtained his training, which made him one of the foremost in this department.

After his return to this country he settled in New York City as a specialist in the diseases of the mouth, throat, nose, larynx, trachea and œsophagus, in a comparatively short time reaching a very high professional standing, and opened a brilliant practical career. He was elected to a professorship in the University of New

York in 1861, and held it for seventeen years. In 1862 the faculty established for him a public clinic, the first in this specialty in the world. Nobody has ever questioned his place in the front rank of Laryngologists, both in America and Europe, as was shown by his election as the first president of the New York Laryngological Society, of which he was the founder, and his ele tion to the first vice-presidency of the International Laryngological Congress at Milano, Italy, in 1881. He published a large number of contributions to Laryngology. He was editor of the Archives of Laryngology up to January 1, 1884, when its publication was discontinued. Numerous lectures and addresses were delivered by him in several of the dental societies of New York, where he demonstrated a new illuminating apparatus for the oral cavity, and spoke of topics concerning oral surgery. His last address was on February 2, before the Medico-Surgical Society of German physicians, explaining the anatomy of the larynx from entirely new points of view, that he never found leisure to publish in the shape of a book.

As a scientist, especially in the department of Biology and Microscopy, he had very few to equal him. He announced in 1872 the theory of the "preservation of plastidules," explaining in the most rational manner the transmission of bodily and mental properties from parents to offspring. The celebrated E. Haeckel, in Jena, discussed the question in his pamphlet on "Perigenesis of the plastidules," which has far less claims for clearness and conciseness than that of Elsberg. He was the first to fully adopt in this country the new doctrine concerning the minute structure of animals and plants, for which he proposed the term "Bioplasson theory." His most elaborate contributions to microscopy, securing him immortality in this department, are his "Researches on the minute structure of the red blood-corpucles," published, 1880, in the annals of the New York Academy of Sciences, and his "Contributions to the minute anatomy of the larynx," published in the Archives of Laryngology, 1882 and 1883.

Since 1864 he was a great sufferer from repeated attacks of periostitis, due to an infection from a patient. Since his sojourn in Aix-la-Chapelle, in Europe, 1881, this trouble seems to have left him. Unfortunately, early in 1883, he again infected himself with diphtheria while performing tracheotomy on a child, and this, though indirectly, caused the fatal result.

He was a man of great energy, devoting hours of night to hard mental labor. His prize essay on tumors of the larynx was written in forty-eight hours, and yet was so accurate that many years later he was not obliged to change his views. Even while suffering from excruciating pains his mind remained active, digesting and discussing philosophical subjects, and studying with zeal the great German Kant. Whenever he made up his mind to carry out an examination he never failed to do it, even though years had elapsed. His failure to publish his standard work on Laryngology, was due to the distressing consciousness that his knowledge of the inflammatory process of the mucous membrane was not sufficient. As a friend no better man could have existed—warm-hearted, generous, always ready to help, and spending many an hour to correct and polish the English of his less gifted friends, suggesting improvements, always spirited, witty, never tired. He married in later years, and enjoyed the company and assistance of a deeply boloved wife and a little daughter, both surviving him.

Louis Elsberg has done his share on this earth. His name shall not perish in the scientific world. Though far advanced in his convictions, often not understood by his fellow-beings, and bitterly attacked for his adherence to novel theories, he never doubted the ultimate victory of these views. Unfortunately he could not witness the triumph of many an idea that he helped to plant. All who knew him mourn his loss. All, moreover, are convinced that his is the wreath of immortality. C. HEITZMANN.

### MENTHOL.

This agent has for some years been employed among the Orientals as a topical application in slight cases of neuralgia, and more recently in this country for a similar purpose. Moulded in small cones, or "pencils," enclosed in boxwood cases, it has been somewhat extensively sold as a "headache cure."

In solution, menthol ʒ ij. with alcohol ℥ i., and applied with a camel's hair brush along the track of a nerve in cases of neuralgia, or even sciatica, produces a grateful and soothing feeling.

So far, no account of its use (to my knowledge), within the oral cavity has been recorded, but Dr. Hungerford, a physician of Stamford, Conn., recently informed me that he had in a single instance applied menthol in solution to the gum to relieve pain from an aching tooth, and with good effect. This remark induced me to test its merits, and I have used it in a number of painful cases of pericementitis and alveolar abscess, with gratifying results. A very small quantity applied to the gum is sufficient. I consider it a valuable addition to our list of therapeutical agents, and one that will come largely into use to allay pain in cases of peridental inflammation. C. E. F.

### FROM RUSSIA.

Dentistry is making advances in all quarters of the world, and travelers in the remote regions of Siberia and "frosty Caucasus," if they cannot have at their command genuine American dentists, may be served with American instruments and appliances. The following is inserted at the request of the editor of the first Russian dental journal ever published. Of his responsibility we know nothing, but the first copy of the "Dentary Revue" is a curiosity. The letters look as if printed upon the other side of the paper, and one involuntarily turns over the leaf to see if they be not showing through.

"I am about to open in St. Petersburg, from the 1st of January, 1885, a central agency of dental goods. I have the honor to solicit the manufacturers to send me their goods, that I may introduce them in Russia, Siberia, the kingdom of Poland, and in the Caucasus.

"The dental art having attained in Russia a considerable degree of development, the want of dental goods has grown very much, therefore I make bold to warrant a good sale, the more as I will insert advertisements about the goods sent in my dental journal 'The Dentary Revue' (Subovrachbng Vestnick), which is the only one existing in Russia.

"My address is, A. P. Sinitzin, Editeur Dentiste, 75–2 Neosky, St. Petersburg, Russia."

### TREATMENT OF CHOLERA.

In view of the expected visit of the cholera to this country during the coming year, any contribution to medical literature, bearing upon the treatment of this disease, should receive careful and earnest consideration on the part of the medical profession.

By the researches of Dr. Koch, it is now known that acids are most useful to kill the cholera microbe, and have been successfully employed by the profession in Europe.

Dr. Chas. Gatchell, of Chicago, in his "Treatment of Cholera," says: "As it is known that the cholera microbe does not flourish in acid solutions; it would be well to slightly acidulate the drinking water. This may be done by adding to each glass of water half a teaspoonful of Horsford's Acid Phosphate. This will not only render the water of an acid reaction, but also render boiled water more agreeable to the taste. It may be sweetened if desired. The Acid Phosphate, taken as recommended, will also tend to invigorate the system and correct debility, thus giving increased power of resistance to disease. It is the acid of the system, a product of the gastric functions, and hence will not create that disturbance liable to follow the use of mineral acids."

The following case is reported from Bangkok, Siam, and may be relied on as authentic: About three months ago a native was attacked with cholera. An American Missionary attended him, and administered all the medicines he could, but at last the man was so far gone that they gave up all hopes of recovery, and would do no more. Relatives of the patient begging the doctor not to give him up as lost, he at last thought of Horsford's Acid Phosphate. After the second dose the patient commenced to revive, and in six hours after, he was pronounced out of danger.

---

### EMENDATORY.

We publish herewith a letter from Dr. Bogue, the substance of which was inadvertently placed without date at the end of his article in this number:

February 14th, 1885.

DEAR DR. BARRETT—Dr. Perry showed me last evening for the first time his improved separators.

I find the separator for the incisors so marked an improvement upon my own form, that I desire to make acknowledgment of its superiority. Yours faithfully,

E. A. BOGUE.

29 East 20th St., New York.

### NOBLESSE OBLIGE.

But the "Noblesse" is not always duly honored. It is not a grateful task to hold up to the world instances of professional degradation, but in the interest of the thousand or so of dental students who are now in near anticipation of their diplomas, we are tempted to publish, *verbatim et literatim*, the following advertisement cut from a newspaper in this State, that they may see how unblushing ignorance and quackery appear to professional eyes. Happily, we hope such graduates are few, and yet more fortunate it is that the late agreement among the colleges will prevent such graduations in the future. This man, holding the diploma of a reputable college, had the advantage of association—for a time, at least—with gentlemen. But he doubtless emerged from a sty, and like the sow that was washed, he has returned to his primeval wallowing. He beautifully illustrates the futility of attempting to make a silver spoon by any patent process of white-washing brass.

DR. BLANKE, D. D. S., DENTAL SURGEON.

Having finished my course in the ——— Dental College I am now qualified to practice over Sergery and Dental Prosthesis in all their branches. From the making and adjusting of an artificial nose to the setting of a broken jaw. Also the Cleoplestic and Khinoplistic operations on Hair lips and Cleft palats, also artificial vorlies attached to plates. Particular attention paid to the preservation of the natural teeth. All work guaranteed and satisfaction given at reasonable rates.

---

### SOUTHERN DENTAL ASSOCIATION.

The Southern Dental Association will hold its seventeenth annual meeting in the City of New Orleans, at Tulane Hall, situated on Dryade Street, between Canal and Common Streets, commencing on the last Tuesday in March.

The Exposition, together with cheap traveling rates, will no doubt induce a large attendance, and the meeting will be one of unusual interest and profit.

J. L. FOUNTAIN,
Cor. Sec., S. D. A.

Bryan, Texas.

### CAPPING EXPOSED PULPS.

In your last issue Dr. W. M. Ramsdell replies to the enquiry of A. B. M. (Dec. '84) concerning exposed pulps. He suggests a cap formed from a portion (I apprehend) of an ordinary lead pencil.

A. B. M. may or may not be satisfied, but should he not be, he may try two forms, both very simple in construction and application, and from their non-conductivity and indestructibility well suited to the purpose.

1st. Soften, in hot water, an ordinary goose-quill. Stamp out with a large sized rubber dam punch a portion, and he will have a cupolated, or dome-formed "cap."

2d. The thin overplus, resulting in vulcanizing a black rubber denture, will also furnish an admirable article, and it can be cupolated by heating a suitable tool and pressing on the disk; apply it to the cavity by means of an ordinary gutta percha solution.

It is unnecessary to remark that this, as all other such operations, should be performed with great care. To this I make no claim of originality, as the whole subject was very fully discussed upward of twenty years since, in the *British Journal of Dental Science*, and the Odontological Society.

LAWRENCE VANDERPANT, L. D. S.,
Orange, New Jersey.

---

### MARRIED.

In Baltimore, Jan. 8th, 1885, Dr. B. M. Wilkerson and Teresa H. Bowie.

Dr. Wilkerson was the founder of this journal, and for some time its responsible editor. Its readers will all join in wishing health, happiness and prosperity to the wedded ones.

---

### DENTAL FURNACE.

Dr. C. H. Land, of Detroit, has invented a new furnace which, it is claimed, possesses decided advantages over any now in existence. It has not yet been fully placed before the profession, but any one who may feel an interest in it can obtain full information by addressing Dr. Land as above.

*To the Editor of the Practitioner:*

The article of Dr. Beers on "A Child's Teeth," reminds me of a similar case which came to my notice. The child, a girl aged about twelve or thirteen, is in very poor health, and anæmic. The mother says she has heart disease, which she inherits from her father. The upper teeth, twenty-eight in number, have thirty-two cavities in them; the upper centrals and laterals are quite destroyed. The lower teeth (number at present forgotten,) have fourteen cavities; the lower front teeth are so far destroyed by approximal decay that they resemble mere pointed pegs. I could give other particulars of the history of the child, but for brevity's sake will not do so now. The whole condition of the case was so bad that I did not believe that I could do any good by attempting to fill them, and with considerable regret told the mother that the child's teeth were not worth the filling. I was impelled to this conclusion not only by the teeth themselves, but by other attendant circumstances.

D. W. BARKER, M. D. S.

---

### DENTAL ASSISTANTS.

In England it is common, when a dental assistant is engaged, to cause him to sign an agreement not to engage in practice in the same neighborhood upon the termination of the engagement. This practice is not usual in this country, and in consequence not unfrequently the dentist suffers by the drawing away of a part of his practice by the man whom he has introduced and recommended to his patients. A case of breach of such an agreement has lately been decided in the English courts.

Drs. Rogers and Cunningham, of Cambridge, engaged as an assistant Mr. Burton. The senior member of the firm subsequently died, and after a time Burton was dismissed, whereupon he opened an office in Cambridge, and commenced independent practice in violation of his agreement. Suit was brought by the surviving partner, and an injunction was granted by the court, prohibiting Burton from practicing as a dentist in Cambridge.

### DENTAL SCHOOL IN AUSTRIA.

It is reported that it is intended to create a chair of dental surgery at the University of Vienna, in connection with an institute in which students of medicine and surgery can become acquainted with everything known about that special branch.

We are convinced that our maxim to allow the practice of dental surgery to physicians only, will be a leading distinction between our proposed institute and those of other countries.—*Oesterreichisch-Ungarische Vierteljahrsschrift fuer Zahnheilkunde.*

---

### AMERICAN MEDICAL ASSOCIATION.

The thirty-sixth annual session will be held in New Orleans, La., on Tuesday, Wednesday, Thursday and Friday, April 28, 29, 30, and May 1, commencing on Tuesday at 11 A. M.

Delegates must receive their appointment from permanently organized State medical societies, and such county and district medical societies as are recognized by representation in their respective State societies, and from the Medical Department of the Army and Navy, and the Marine Hospital service of the United States.

---

### NATIONAL ASSOCIATION OF DENTAL EXAMINERS.

The third meeting of the National Association of Dental Examiners will be held at New Orleans, on Tuesday, March 31, 1885.

All State Boards not already members of this association are earnestly invited to send representatives to this meeting.

GEORGE H. CUSHING,

Secretary.

---

### STATE BOARD OF MEDICAL EXAMINERS.

At the late meeting of the New York State Medical Society, a draft of a bill was presented and unanimously concurred in for the establishment of a State board of medical examiners, and making a State examination necessary hereafter to a license for the practice of medicine.

### EXTRACTION OF TEETH.

Dr. N. J. Hepburn, of New York, communicates to us the following method for the painless extraction of teeth. It has been used in some portions of Europe for a long time, with great satisfaction:

Dilute the tincture of Cannabis Indica made from the purified extract, with from three to five parts of warm water. Rub this over the gums with the finger for a short time, and then dip the warmed beak of the forceps in it before their application to the tooth.

---

### JURY DUTY.

Dentists are frequently annoyed by being summoned to serve upon juries. Especially is this the case in the smaller towns. One who had thus been troubled has taken the pains to look up the law upon the subject, and desires to communicate the information to his professional brethren. He finds that by Chap. 358 of the Laws of the State of New York for 1881, dentists are exempt from jury duty. The judge sitting in the case upon which he was summoned refused to excuse him until shown the statute, the existence of which was previously unknown to him.

---

### CHLOROFORM AND THE FIFTH PAIR OF NERVES.

W. E. Buck, physician to the Leicester Infirmary, says, in the London *Lancet*, that if a patient be not thoroughly under the influence of chloroform, any irritation of the fifth nerve will produce slowing of the heart's action and final stoppage through the pneumogostic nerves. He thinks this may account for many deaths in dentists' chairs, and advises that in any operation involving the fifth nerve the anæsthesia should be pushed until the patient is completely under its influence.

(47.) Dentists who practice in towns without water supply and gas, have to depend on small steam motors to run their dental machinery. Will some one having knowledge and experience in these, inform us as to the best to adopt, considering power, noise, cost (price and running expense), and steam producing apparatus, etc.? B. H. T., South Carolina.

(48.) It has been said by some parties who have applied cocaine to sensitive teeth, that it prevents pain in excavating. Others who have tried it repeatedly, say that it has no such effect. How can we account for such contradictory statements? A contributor in your February number asks how cocaine acts on animal tissue. I would like to know how it acts on the *hard* tissue. W.

(49.) Will some one please explain the term "knuckling," as used by Drs. Brown and Atkinson in the February PRACTITIONER. B.

(50.) Years ago it was the practice to fill cavities in devitalized teeth without treatment, and then beneath the gum to drill a hole to the pulp cavity, to permit drainage. Such teeth, sometimes with a continuous discharge of pus, occasionally fall into my hands now. What should be done with them? MODERN DENTIST.

### Answers.

R. (42.) To remove a Swiss broach broken off in the root, pack the canal with chloride of sodium (common salt); let it remain three or four days; then open, and the broach will come out without any difficulty. D. D. S.

S. B. (44.) Twenty States have laws regulating the practice of dentistry. For full information on this subject see *Caulk's Dental Annual* for 1884–85, page 40. D. D. S.

LABORATORY. (43.) You are wrong in advising a "clean sweep," especially for the lower jaw. If there are any teeth or roots that are or can be put in a healthy condition, do not extract them, as they are worth millions to the patient, in preventing lower plates from "swinging around loose." When you make a "clean sweep" you may be able to make a more beautiful set, but they will not be half so good for actual use. S.

STUDENT. (41.) Melt your waste amalgam, old fillings, etc., in a clean crucible, and as soon as melted pour into an ingot. File with a coarse file; remove the iron with magnet, and the new alloy is ready for use. Flagg on plastic materials is as good as anything you can find for instruction. S. S.

Thanks to "M. D." in February number for his reply to query 38. The patient has not made her appearance since, but when she comes again the suggestions kindly given will be well considered, and may aid materially in diagnosing the case. BOSTON.

R. (42.) Carry into the root canal a fiber of cotton saturated with tincture of iodine, and allow it to remain a few days properly sealed. The iodine will destroy the broach. G.

LABORATORY. (43.) As a general rule, where there are only two or three teeth remaining it is best to remove them, as a better articulation may thus be secured, as well as a much better fitting plate. The durability of such teeth, if allowed to remain, is very doubtful. If, however, the teeth are in the front of the mouth, or include the cuspids, it may be well to retain them for a while. B.

P. B. R. (45.) Sets quicker. "This and nothing more." S.

Several communications are necessarily held over.—EDITOR.

